# Eating behavior in patients with smell loss

**DOI:** 10.3389/fnut.2022.993639

**Published:** 2022-11-09

**Authors:** David T. Liu, Bernhard Prem, Gunjan Sharma, Julia Kaiser, Gerold Besser, Christian A. Mueller

**Affiliations:** Department of Otorhinolaryngology, Head and Neck Surgery, Medical University of Vienna, Vienna, Austria

**Keywords:** anosmia, hyposmia, eating behavior, quality of life, smell loss, olfactory and gustatory system

## Abstract

**Background:**

The objective of this study was to determine how clinical characteristics and validated quality of life (QoL)-measures are associated with eating behavior in patients with olfactory dysfunction (OD).

**Methods:**

For this cross-sectional study, 150 OD patients of different causes were retrospectively recruited. Olfactory function was measured using the Sniffin’ Sticks (TDI), while olfactory-related QoL was evaluated with the Questionnaire of OD negative and positive statements (QOD-NS and QOD-PS). The importance of olfaction was measured using the Importance of Olfaction Questionnaire (IOQ). The Dutch Eating Behavior Questionnaire (DEBQ) assessed eating behavior based on emotional, external, and restrained eating. Associations were sought between eating behavior metrics (as dependent variables) with clinical characteristics and olfactory-related outcome measures.

**Results:**

Emotional, external, and restrained eating behavior deviating from normative standards were reported in 54%, 71.3%, and 68% of patients, respectively. Multivariate regression modeling revealed that emotional eating was associated with age (ß = –0.227, *p* = 0.032), the body mass index (BMI, ß = 0.253, *p* = 0.005), the TDI (ß = 0.190, *p* = 0.046), and the QOD-NS (ß = 0.203, *p* = 0.049). External eating was associated with OD duration (ß = 0.291, *p* = 0.005), the TDI (ß = 0.225, *p* = 0.018), the QOD-PS (ß = –0.282, *p* = 0.008), and the IOQ (ß = 0.277, *p* = 0.004). Restrained eating was associated with age (ß = 0.216, *p* = 0.033), the BMI (ß = 0.257, *p* = 0.003), male gender (ß = –0.263, *p* = 0.002), and the IOQ (ß = 0.332, *p* < 0.001).

**Conclusion:**

Clinical characteristics and olfactory outcome measures differentially impact eating styles in OD patients. Our study’s results highlight the importance of considering unfavorable changes in eating behavior during clinical counseling.

## Introduction

Human flavor perception involves multiple senses, including the sense of vision, taste, smell, and hearing (1). Among those senses, the olfactory system, especially retronasal olfaction (i.e., odor molecules that reach the olfactory epithelium during expiration when eating and drinking), plays a crucial and significant role. Therefore, it is unsurprising that olfactory dysfunction (OD) alters flavor perception and quality of life (QoL) in affected individuals (2–4). Indeed, previous studies provided evidence that smell loss adversely influences dietary intake, which may lead to severe complications in terms of nutritional risk (5–7). While previous studies also showed that patients with OD report eating behavior changes resulting in weight gain and loss (7–9), its associations with disease-specific variables or olfactory-related QoL measures have yet to be evaluated. Identifying relevant associations is crucial to understand further the critical drivers of adverse eating behavior and to develop strategies to overcome OD-related behavioral changes.

Changes in dietary behavior and macronutrient intake are frequently reported in patients with smell loss (8, 10). Indeed, it has been shown that patients reported preferring more salty and spicy dishes since the onset of smell loss (5, 11). Previous studies also showed that weight gain occurs more frequently in younger patients with OD (5). Being overweight is believed to result from a chronic positive energy balance strongly associated with food intake and eating behavior (12). From a psychological standpoint, multiple theories have been proposed to explain eating behavior associated with obesity, such as emotional eating (13) (i.e., eating to deal with emotional arousal instead of satisfying hunger), external eating (14) (i.e., eating in response to external food cues), and restrained eating (15) (i.e., restrictive eating to regulate body weight). The Dutch Eating Behavior Questionnaire (DEBQ), a validated and high-quality patient-reported outcome (PRO), measures the eating traits mentioned above and has been translated into various languages (16–18).

Smell loss can result from various causes and is associated with a significant loss of information (19, 20). Despite novel treatment strategies for OD, only a small proportion of patients regain normal function over time (21). Although OD disrupts various areas in daily life, loss of taste and flavor perception is one of the most dominant drivers of decreased QoL in affected individuals (2). Highlighting the critical association between flavor perception and smell loss, previous studies have shown that OD leads to significant changes in eating behavior (8, 10). These findings suggested that specific clinical characteristics are differentially associated with eating behavior in OD patients. However, no study has examined the eating behavior in smell loss patients and its associations with disease-specific and OD-related PROs.

To further understand OD patients’ eating behavior changes, we must first identify which clinically relevant variables or QoL measures are the primary drivers. In this study, we first aimed to (i) compare emotional, external, and restrained eating behavior in patients with OD against normative results from a culturally similar, healthy population. We hypothesized that the eating behavior of smell loss patients differs significantly from that of a healthy population. Secondly, we aimed to (ii) explore differences in emotional, external, and restrained eating between various causes of smell loss. We hypothesized that the reason for smell loss affects different eating traits in OD patients. Thirdly, we aimed to analyze the associations between demographics (age, gender, body mass index—BMI), OD-related variables (such as reason and measured olfactory function), and olfactory-related PROs, the Questionnaire of OD, QOD (measuring the burden and coping strategies of patients with OD) (22), and the Importance of Olfaction Questionnaire, IOQ (measuring the individual importance of olfaction) (23) with (iii) the emotional, (iv) external, and (v) restrained eating behavior in a cohort of OD patients with different causes. We hypothesized that demographics, olfactory-related variables (such as the measured olfactory function), and olfactory-related PROs differentially impact the emotional, external, and restrained eating behavior.

## Materials and methods

### Patient selection

This study was approved by the Ethical Scientific Committee of the Medical University of Vienna (1984/2021). We retrospectively identified patients that were referred to the Smell and Taste Clinic of the Department of Otorhinolaryngology, Head and Neck Surgery of the Medical University of Vienna with subjective smell loss. Data were collected from each patient’s visit to our clinics for the management of smell loss between 04/2019 and 10/2021. We excluded patients that did not complete the studied PROs described below.

### Data collection

Data collected at the time of each patient’s visit included age, gender, self-reported weight and height, suspected underlying etiology of smell loss according to the “Position paper of OD”(19), duration of OD, and quantitative olfactory function measured using the three subtests Sniffin’ Sticks Threshold, Discrimination, and Identification Test (TDI) (24, 25). The BMI was calculated as weight in kg/[(height in m^2^)]. Patients categorized as coronavirus-19 (COVID-19) related smell loss were either proven by polymerase chain reaction (PCR) during acute sickness or based on blood samples positive for antibodies against SARS-CoV-2 (before the availability of COVID-19 vaccines). Quantitative olfactory function was classified based on normative data available for the Sniffin’ Sticks Test: (a) normal olfactory function, normosmia: TDI score greater than or equal 30.75, (b) reduced olfactory function, hyposmia: TDI score lower than 30.75 and greater than 16, and (c) complete loss of olfactory function, anosmia: TDI score lower than or equal 16 (26, 27).

All patients completed the validated German version of the DEBQ, a high-quality and widely used 30-item PRO that evaluates eating behavior contributing to weight gain based on three subdomains with 10 items each: emotional eating (i.e., “desire to eat when depressed or discouraged”), external eating (i.e., “eating more than usual when the food smells and looks good”), and restrained eating (i.e., “watching exactly what you eat”). All items are scored on a 5-point Likert scale ranging from 1-“never” to 5-“very often” (16, 18). The average score within each subdomain can be compared against large normative datasets. A higher score represents more problems related to weight gain within the subdomain in question (16–18). Each patient also completed the Questionnaire of Olfactory Dysfunction (QOD), a validated and widely used PRO that evaluates disease-specific QoL in patients with smell loss (2, 22). The QOD consists of two subsections, the 17-item QOD-negative statement (QOD-NS), which evaluates the negative impact of OD on QoL, and the 2-item QOD-positive statement (QOD-PS), which evaluates the ability of OD patients to cope with smell loss (2, 28). The QOD is scored on an integer scale ranging from 0-“I disagree” to 3-“I agree.” Answers are summed with a higher QOD-NS score representing lower olfactory-related QoL, while a higher QOD-PS score represents better coping abilities. The Importance of Olfaction Questionnaire (IOQ) measured the individual significance of olfaction (23). The IOQ is a 20-item PRO based on Likert-scale items ranging from 0-“I totally disagree” to 3-“I agree.” Answers from IOQ-items are summed with a higher IOQ score representing a higher significance of olfaction to affected individuals.

### Statistical analysis

Statistical analyses and data visualization were performed under SPSS 26.0 (Chicago, IL, USA) and GraphPad Prism 9.1.0 (GraphPad Software, Inc., La Jolla, CA). To test for differences in eating behavior in our OD patients, we used a one-way analysis of variance (ANOVA) followed by Tukey’s *post hoc* test. Univariate and multivariate linear regression analyses were used to associate emotional, restrained, and external eating behavior (as dependent variables) with age, gender (reference: female), BMI, duration of smell loss (in months), the reason for smell loss (postinfectious- PIOD, posttraumatic, idiopathic, and COVID-19 OD, reference: PIOD), quantitative olfactory function (Sniffin’ Sticks TDI score), the burden of smell loss as QOD-NS, coping strategies as QOD-PS, and the individual importance of olfaction as IOQ. A *p*-value < 0.05 was considered statistically significant.

## Results

### Characteristics of study subjects

This study included 150 patients that visited our outpatient department with the main complaint of smell loss (62.5% female, mean age ± *SD* = 47.8 ± 18.8 years). The reason for smell loss included: COVID-19 related (*n* = 46), idiopathic (*n* = 37), PIOD (*n* = 29), posttraumatic (*n* = 14), sinonasal (*n* = 9), iatrogenic following neurosurgical procedures (*n* = 6), toxic (*n* = 4), congenital (*n* = 3), and neurodegenerative (*n* = 3). The mean duration of smell loss was 35.4 months (*SD* = 46.8). Olfactory testing using the Sniffin’ Sticks (TDI) showed that the biggest group of our patients was hyposmic (*n* = 89), followed by 46 anosmic and 15 normosmic patients with subjective OD (Table 1).

**TABLE 1:** Demographics and clinical characteristics.

Patients with smell loss (*n* = 150)	
Age in years, mean (SD)	46.8 (18.6)
Gender (N)	92F, 58M
Duration of smell loss in months, mean (SD)	35.4 (46.8)
Body mass index (BMI), mean (SD)	25.6 (5.5)
BMI < 30	126 (84%)
BMI > 30	24 (16%)
**Olfactory function**	
Sniffin’ sticks TDI score, mean (SD)	21.0 (8.2)
Threshold, mean (SD)	3.7 (2.4)
Discrimination, mean (SD)	9.2 (3.2)
Identification, mean (SD)	8.0 (4.0)
Olfactory function	
Normosmics	15 (10.0%)
Hyposmic	89 (59.3%)
Anosmic	46 (30.7%)
**Reason for smell loss**	
COVID-19	46 (30.7%)
Idiopathic	37 (24.7%)
Postinfectious	29 (19.3%)
Posttraumatic	14 (9.3%)
Sinonasal	9 (6.0%)
Iatrogen	6 (4.0%)
Toxic	4 (2.7%)
Congenital	3 (2.0%)
Neurodegenerative	3 (2.0%)
**Patient-reported outcome measures**	
QOD-NS, mean (SD)	18.1 (11.4)
QOD-PS, mean (SD)	3.6 (2.0)
IOQ, mean (SD)	32.3 (13.5)
Emotional eating behavior, mean (SD)	2.0 (0.8)
External eating behavior, mean (SD)	2.9 (0.7)
Restrained eating behavior, mean (SD)	2.7 (0.9)

Continuous data are presented as mean (standard deviation). Categorical data are presented as numbers (%).

### Eating behavior in patients with smell loss of different causes

We were first interested in knowing whether the eating behavior of our OD patients differed from that of the general population. We, therefore, compared results from the three DEBQ subdomains (i.e., emotional, external, and restrained eating behavior) against normative means derived from a culturally similar cohort (16).

We found that 107 patients (71.3%) scored above the normative mean for external eating behavior, while 102 (68%) scored above the normative mean for restrained eating behavior. Similarly, we found that 81 patients (54%) scored above the normative mean regarding emotional eating.

We were then interested to know whether eating behavior—represented by emotional, external, and restrained eating—differed between different etiology groups. We, therefore, compared results from the DEBQ between our COVID-19, idiopathic, posttraumatic, and PIOD groups. We excluded other causes of smell loss due to the small group sizes.

**FIGURE 1:**
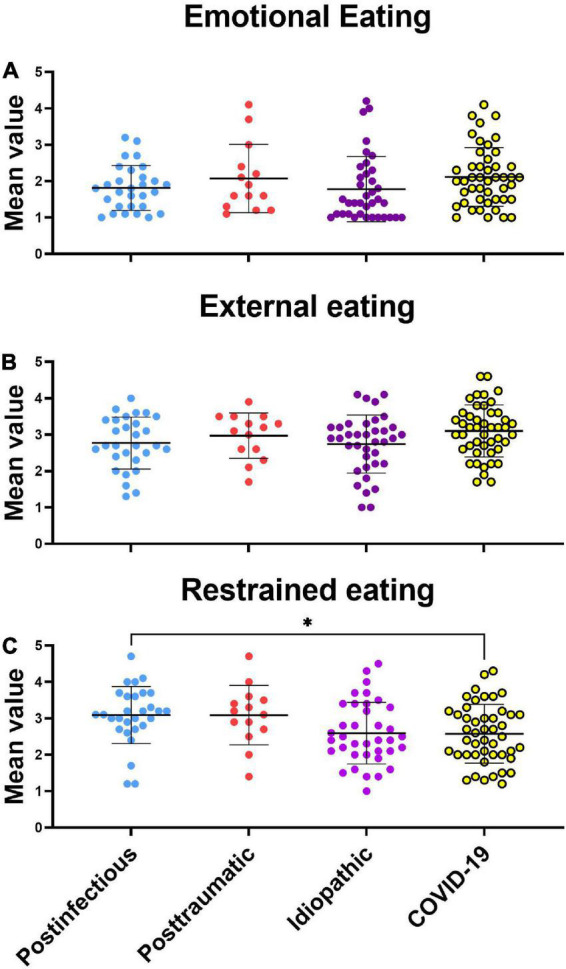
Scattergram (mean ± SD) of the Dutch eating behavior questionnaire by different eating styles. Groups were compared by the ANOVA test with *post hoc* Tukey’s test. *P < 0.05. **(A)** Emotional eating style, **(B)** external eating style, **(C)** restrained eating style.

One-way ANOVA revealed no significant difference in emotional eating behavior between the PIOD, posttraumatic, idiopathic, and COVID-19 OD groups [*F*(3, 122) = 1,547, *p* = 0.2058]. Similarly, one-way ANOVA revealed no significant difference in external eating behavior between different smell loss groups [*F*(3, 122) = 2.089, *p* = 0.1052]. On the contrary, one-way ANOVA revealed significant differences in restrained eating behavior between OD groups [*F*(3, 122) = 3.638, *p* = 0.0148]. Tukey’s *post hoc* test revealed that PIOD patients scored significantly higher on the restrained eating behavior scale than COVID-19 OD patients (*p* = 0.042) (Figure 1).

### Age, body mass index, measured olfactory function, and olfactory-related quality of life are associated with emotional eating behavior

As we found that the reason for smell loss might be associated with eating behavior, we were next interested in knowing which factors were associated with the emotional eating behavior in our OD patients. We performed univariate and multivariate linear regression analyses with the outcome of emotional eating behavior. We included demographics, disease-specific variables, and olfactory-related PROs (QOD-NS, QOD-PS, and IOQ) as explanatory variables. We also performed multivariate regression modeling excluding olfactory-related PROs to report clinically more relevant results as there are differences in olfactory outcome measures used between clinics worldwide.

**FIGURE 2:**
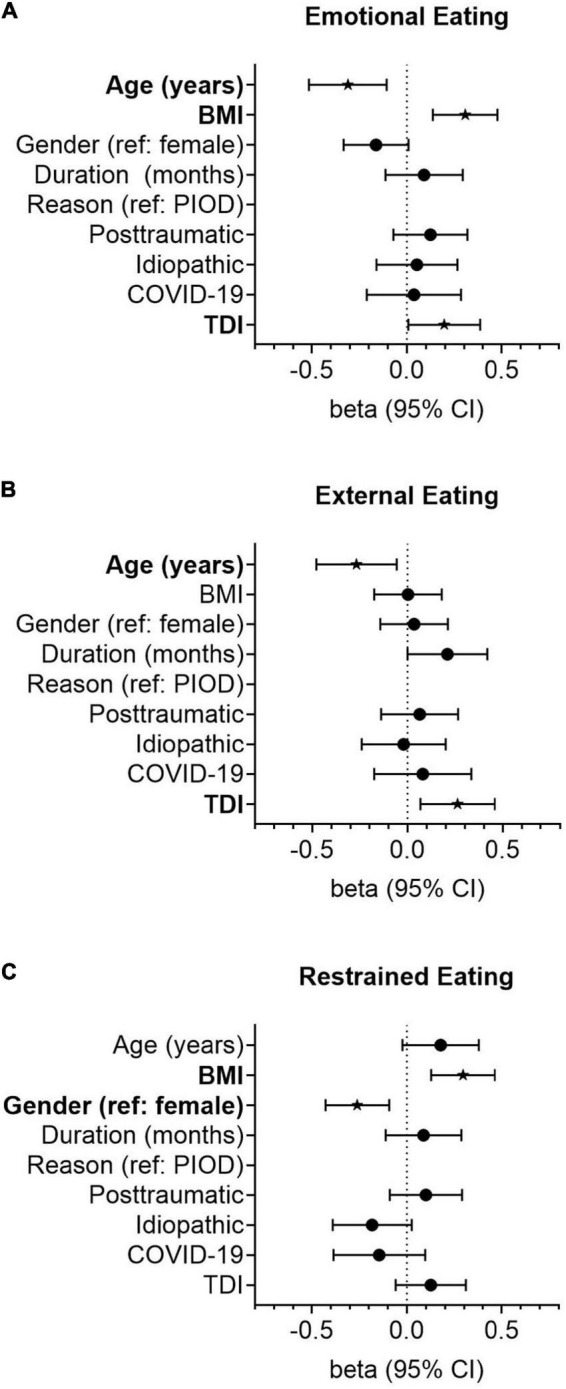
Forest plots showing the association of smell loss-related variables with the emotional **(A)**, external **(B)**, and restrained **(C)** eating styles. Odds ratios (OR) were calculated using linear regression models adjusted for age (years), gender (reference: female), duration of smell loss (month), the reason for smell loss (reference: postinfectious smell loss), and quantitative olfactory function (TDI). Points represent group-specific OR point estimates, and lines indicate the respective 95% confidence interval (CI).

Univariate linear regression analysis revealed that age (ß = –0.294, *p* = 0.001) and the QOD-PS (ß = –0.218, *p* = 0.014) were negatively associated with emotional eating. In contrast, the BMI (ß = 0.182, *p* = 0.041), TDI score (ß = 0.203, *p* = 0.023), QOD-NS (ß = 0.376, *p* < 0.001), and the IOQ (ß = 0.325, *p* < 0.001) were significantly positively associated with emotional eating behavior. In multivariate regression modeling, we found that age (ß = –0.227, *p* = 0.032), BMI (ß = 0.253, *p* = 0.005), the TDI score (ß = 0.190, *p* = 0.046), and the QOD-NS (ß = 0.203, *p* = 0.049) remained significantly associated (Table 2). When omitting olfactory-related QoL measures (i.e., the QOD-NS, QOD-PS, and IOQ) from multivariate regression modeling, age (ß = –0.311, *p* = 0.003), BMI (ß = 0.3072, *p* = 0.001), and the TDI score (ß = 0.196, *p* = 0.042) remained significantly associated with emotional eating behavior in our patients (Figure 2).

**TABLE 2:** Associations with emotional eating.

	Univariate linear regression analysis		Multivariate regression modeling	
		
	β (95% CI)	*P*-value	β (95% CI)	*P*-value
Age (years)	**–0.294 (–0.464 to –0.124)**	**0.001**	**–0.227 (–0.435 to –0.020)**	**0.032**
BMI	**0.182 (0.007–0.357)**	**0.041**	**0.253 (0.079–0.427)**	**0.005**
Gender (female)	**–0.180 (–0.355 to –0.005)**	**0.044**	–0.119 (–0.290 to 0.053)	0.172
Duration of smell loss (months)	–0.095 (–0.272 to 0.082)	0.291	0.131 (–0.070 to 0.332)	0.199
Etiology of smell loss				
Postinfectious	Reference	—	Reference	—
Posttraumatic	0.056 (–0.122 to 0.233)	0.534	0.046 (–0.149 to 0.240)	0.643
Idiopathic	–0.128 (–0.305 to 0.048)	0.152	0.016 (–0.192 to 0.225)	0.876
COVID-19	0.161 (–0.015 to 0.336)	0.072	–0.019 (–0.264 to 0.225)	0.875
TDI	**0.203 (0.029–0.377)**	**0.023**	**0.190 (0.003–0.377)**	**0.046**
QOD-NS	**0.376 (0.211–0.540)**	**<0.001**	**0.203 (0.001–0.406)**	**0.049**
QOD-PS	**–0.218 (–0.391 to –0.044)**	**0.014**	–0.017 (–0.225 to 0.192)	0.876
IOQ	**0.325 (0.157–0.493)**	**<0.001**	0.133 (–0.057 to 0.322)	0.168

β, Linear regression coefficient. Bold values denote statistical significance at the *p* < 0.05 level.

### Duration of smell loss, measured olfactory function, the ability to cope with smell loss, and the individual importance of olfaction are associated with external eating behavior

In the next step, we were interested to know which factors were associated with external eating. We, therefore, also performed univariate and multivariate linear regression analyses with the outcome of external eating and included demographics, disease-specific information, and olfactory-related QoL measures as explanatory variables.

In univariate linear regression analysis, we found that age (ß = –0.308, *p* < 0.001) and the QOD-PS (ß = –0.347, *p* < 0.001) were significantly negatively associated with external eating, while COVID-19-related smell loss (reference: PIOD, ß = 0.202, *p* = 0.024), the TDI score (ß = 0.260, *p* = 0.003), the QOD-NS (ß = 0.221, *p* = 0.013), and the IOQ (ß = 0.388, *p* < 0.001) were positively associated with the external eating behavior in smell loss patients. In multivariate regression modeling, we found that the duration of smell loss (ß = 0.291, *p* = 0.005), the TDI score (ß = 0.225, *p* = 0.018), the QOD-PS (ß = –0.282, *p* = 0.008), and the IOQ (ß = 0.277, *p* = 0.004) remained significantly associated (Table 3). When omitting olfactory-related QoL measures from the analysis, we found that age (ß = –0.267, *p* = 0.014) and the TDI score (ß = 0.263, *p* = 0.009) remained significantly associated with the external eating behavior in OD patients (Figure 2).

**TABLE 3:** Associations with external eating.

	Univariate linear regression analysis		Multivariate regression modeling	
		
	β (95% CI)	*P*-value	β (95% CI)	*P*-value
Age (years)	**–0.308 (–0.477 to –0.139)**	**<0.001**	–0.144 (–0.350 to 0.062)	0.169
BMI	–0.097 (–0.274 to 0.079)	0.278	0.014 (–0.159 to 0.187)	0.874
Gender (female)	–0.049 (–0.226 to 0.129)	0.589	0.107 (–0.063 to 0.277)	0.216
Duration of smell loss (months)	–0.039 (–0.217 to 0.139)	0.665	**0.291 (0.091–0.491)**	**0.005**
Etiology of smell loss				
Postinfectious	Reference	—	Reference	—
Posttraumatic	0.032 (–0.145 to 0.210)	0.719	–0.012 (–0.205 to 0.182)	0.906
Idiopathic	–0.143 (–0.319 to 0.033)	0.110	–0.053 (–0.260 to 0.154)	0.612
COVID-19	**0.202 (0.027–0.376)**	**0.024**	–0.020 (–0.263 to 0.223)	0.870
TDI	**0.260 (0.088–0.432)**	**0.003**	**0.225 (0.040–0.411)**	**0.018**
QOD-NS	**0.221 (0.047–0.394)**	**0.013**	–0.013 (–0.215 to 0.188)	0.897
QOD-PS	**–0.347 (–0.514 to –0.180)**	**<0.001**	**–0.282 (–0.489 to –0.074)**	**0.008**
IOQ	**0.388 (0.224–0.552)**	**<0.001**	**0.277 (0.089–0.465)**	**0.004**

β, Linear regression coefficient. Bold values denote statistical significance at the *p* < 0.05 level.

### Age, body mass index, gender, and the individual importance of olfaction are associated with restrained eating behavior

In the last step, we wanted to identify factors associated with restrained eating behavior. We, therefore, performed univariate and multivariate linear regression analyses with restrained eating behavior as the outcome variable and demographics, disease-specific information, and olfactory-related QoL measures as explanatory variables.

In univariate linear regression analysis, we found that age (ß = 0.220, *p* = 0.013), BMI (ß = 0.315, *p* < 0.001), and IOQ (ß = 0.243, *p* = 0.006) were positively associated with restrained eating. In multivariate regression modeling, we found that age (ß = 0.216, *p* = 0.033), BMI (ß = 0.257, *p* = 0.003), gender (reference: female, ß = –0.263, *p* = 0.002), and the IOQ (ß = 0.332, *p* < 0.001) were significantly associated with the restrained eating behavior in smell loss patients (Table 4). When omitting olfactory-related QoL measures from multivariate regression modeling, we found that gender (ß = –0.261, *p* = 0.003) and BMI (ß = 0.295, *p* = 0.001) remained significantly associated with restrained eating (Figure 2).

**TABLE 4:** Associations with restrained eating.

	Univariate linear regression analysis		Multivariate regression modeling	
		
	β (95% CI)	*P*-value	β (95% CI)	*P*-value
Age (years)	**0.220 (0.046–0.393)**	**0.013**	**0.216 (0.018–0.413)**	**0.033**
BMI	**0.315 (0.146–0.484)**	**<0.001**	**0.257 (0.091–0.423)**	**0.003**
Gender (female)	–0.169 (–0.344 to 0.006)	0.058	**–0.263 (–0.427 to -0.100)**	**0.002**
Duration of smell loss (months)	0.106 (–0.071 to 0.283)	0.238	0.085 (–0.107 to 0.277)	0.382
Etiology of smell loss				
Postinfectious	Reference	—	Reference	—
Posttraumatic	0.140 (–0.036 to 0.316)	0.119	0.038 (–0.148 to 0.223)	0.687
Idiopathic	–0.124 (–0.300 to 0.052)	0.166	–0.193 (–0.391 to 0.006)	0.057
COVID-19	–0.165 (–0.340 to 0.011)	0.065	–0.154 (–0.387 to 0.079)	0.192
TDI	0.007 (–0.171 to 0.184)	0.942	0.070 (–0.108 to 0.248)	0.440
QOD-NS	0.112 (–0.064 to 0.289)	0.211	0.031 (–0.163 to 0.224)	0.753
QOD-PS	0.160 (–0.016 to 0.335)	0.074	0.154 (–0.045 to 0.354)	0.128
IOQ	**0.243 (0.071–0.415)**	**0.006**	**0.332 (0.152–0.513)**	**<0.001**

β, Linear regression coefficient. Bold values denote statistical significance at the *p* < 0.05 level.

## Discussion

This study sought to understand further the relationship between clinical characteristics (such as reason and duration of smell loss) and olfactory-related PROs (QOD and IOQ) with different eating behavior theories (i.e., external, emotional, and restrained eating). We found that emotional, restrained, and external eating behavior problems are frequently reported in OD patients. Furthermore, we showed that clinical characteristics and PROs are differentially associated with the three eating behavior theories. We found that age, BMI, quantitative olfactory function (TDI), and the negative impact of smell loss on daily life (QOD-NS) were significantly associated with emotional eating. Similarly, we found that age, BMI, gender, and the individual importance of olfaction (IOQ) were associated with restrained eating. Likewise, we found that the duration of OD, quantitative olfactory function (TDI), coping abilities (QOD-PS), and the individual importance of olfaction (IOQ) were significantly associated with external eating.

Investigating OD patients’ emotional eating behavior may be particularly important because previous work has shown that emotional eating contributes more to weight outcomes than external eating. Indeed, studies have shown that 60% or more of individuals who struggle with weight are emotional eaters (29, 30). Furthermore, longitudinal studies have also shown that higher levels of emotional eating predict more significant weight gain (31). Our study found that the BMI, quantitative olfactory function (TDI), and the olfactory-related QoL (QOD-NS) were positively associated with emotional eating behavior, while age was negatively associated. Our finding of an association between olfactory-related QoL and emotional eating was not surprising, considering that emotional eating is usually defined as overeating in response to negative emotions. Therefore, a lower olfactory-specific QoL may also impact emotional functioning, resulting in more significant problems related to emotional eating behavior in OD patients. Another interesting finding was the positive association between the TDI score and emotional eating behavior. One explanation for this association might be related to the significant role of olfaction in human flavor perception. Previous studies have shown that hyperpalatable food might serve as “comfort food” and as a form of self-medication (32, 33). Furthermore, it has also been proposed that emotional eaters are more engaged in food consumption to achieve short-term gratification from negative feelings (32). It might therefore be speculated that patients with a decreased or an absent olfactory function (i.e., anosmics and severe hyposmic patients) are unable to experience the full breadth of flavor during food consumption and therefore also do not achieve the same short-term gratification compared to their normosmic counterparts, resulting in less emotional eating behavior.

Our results from associations with external eating revealed that the duration of smell loss, measured olfactory function (TDI), the ability to cope with smell loss (QOD-PS), and the individual importance of olfaction (IOQ) were significantly associated. Regarding the duration of smell loss, it might be hypothesized that patients with a longer duration of smell loss might rely more on the other senses (such as the sense of vision) while eating and drinking, thus might be triggered more easily to eat more than usual when the food simply looks appetizing. Similarly, the finding of a positive association between the measured olfactory function and the external eating behavior was not surprising since elevated responsiveness to food-related odor cues in the immediate environment might explain why patients with a higher olfactory function have more problems related to the external eating behavior. Previous studies have shown that, it is assumed that the functionality of coping strategies depends strongly on the repertoire available that would allow patients to respond to specific challenges, such as stopping eating when the food smells and looks good (34). One might hypothesize that those with fewer strategies and abilities also have significantly more problems related to external eating behavior, which we found to be the case. Similarly, one might also hypothesize that patients with higher individual importance of olfaction try to use their sense of smell and flavor more in daily life, which is why they also face more difficulties in stopping eating and drinking in cases the food smells and looks good.

As mentioned above, BMI, gender, and the individual importance of olfaction (IOQ) were associated with restrained eating behavior. While we found a positive association between age, BMI, and the IOQ with restrained eating behavior, the male gender was negatively associated. Our finding of an association between higher age and more restrained eating behavior has also been reported previously. The authors assessed the eating behavior of students aged 18–50 and found that older students were more likely to restrain their eating behavior. The authors explained that younger adults might be less enthusiastic about changes in dietary behavior, although they might be more concerned about their health (35). This is supported by previous studies showing that food choices change with age. Older adults consume less energy-dense food and eat more energy-diluted grains and vegetables (36). Similarly, previous work has shown that higher BMI predicts more restrained eating among adolescents (37). Interestingly, the same study also showed that although higher BMI predicted restrained eating, restrained eating did not predict weight loss simultaneously. We, therefore, hypothesized that BMI might also be positively associated with restrained eating in OD patients, which we found to be the case. Interestingly, we also found that the IOQ was significantly associated with restrained eating, indicating that the higher the individual importance of olfaction, the higher the restrained eating behavior. One explanation of this finding might relate to the olfactory subdimensions that the IOQ questionnaire evaluates. While the application-subscale assesses how patients use their sense of smell daily, the consequence-subscale evaluates conclusions drawn from olfactory perceptions. As the sense of smell contributes significantly to flavor perception (1, 38), one might hypothesize that smell loss patient’s detriment in flavor perceptions also limits the extent to which patients use their sense of smell or draw conclusions from it in daily life, thus naturally limiting the need for restrained eating behavior. Lastly, our finding of sex differences in restrained eating was not unexpected, as previous studies provided evidence that women report weight control behaviors more frequently than men (39). Furthermore, it has also been shown that girls consistently score higher values in restrained eating than boys (40), from childhood to adolescence (37).

Regarding the clinical relevance of our findings, results might be implemented easily into the clinical management of patients with smell loss. The potential benefits gained when patients are informed about or referred to interventions to change dietary behavior should be considered during counseling. Furthermore, consulting patients about the possibilities of compensating for the loss of flavor perception with supportive treatment options such as flavor enhancement may help maintain food intake and behavior (41, 42). Similarly, emphasizing the importance of treatment adherence to olfactory training, the recommended treatment option for most causes of smell loss might improve olfactory function and changes in eating behavior associated with smell loss (43, 44).

It is important to mention that although we included a large cohort of smell loss patients with different causes, our results should be interpreted with caution. Most importantly, this cross-sectional design did not demonstrate any causative mechanisms. Future studies should seek to identify changes in the eating behavior of OD patients that recover clinically relevantly. Secondly, this study used data collected retrospectively and from a single center and is associated with all limitations inherited from this study design. However, we believe that our findings illustrate that smell loss is frequently associated with changes in eating behavior and that our results highlight the importance of unraveling key drivers of adverse eating behavior and developing strategies to overcome smell loss-related behavioral changes.

In summary, this study showed that alterations in eating behavior are frequently reported in patients with smell loss. Furthermore, we also found that clinical characteristics and olfactory-related PROs differentially impact eating styles in OD patients. Our study’s results highlight the importance of considering unfavorable changes in eating behavior during clinical counseling.

## Data availability statement

The raw data supporting the conclusions of this article will be made available by the authors, without undue reservation.

## Ethics statement

This study was approved by the Ethical Scientific Committee of the Medical University of Vienna (1984/2021). Written informed consent for participation was not required for this study in accordance with the national legislation and the institutional requirements.

## Author contributions

DL: concept of study, collection of data, analysis of results, write up of manuscript, and critical review of all contents. BP, JK, GS, GB, and CM: concept of study, collection of data, and critical review of all contents. All authors contributed to the article and approved the submitted version.
